# A review of the health and economic implications of patent protection, with a specific focus on Thailand

**DOI:** 10.1186/1478-4505-10-24

**Published:** 2012-08-01

**Authors:** Inthira Yamabhai, Richard D Smith

**Affiliations:** 1 Health Intervention and Technology Assessment Program (HITAP), Ministry of Public Health, Nonthaburi, Thailand; 2 Department of Global Health and Development, Faculty of Public Health & Policy, London School of Hygiene & Tropical Medicine, London, UK

## Abstract

**Background:**

Although it has been two decades since the Thai Patent Act was amended to comply with the Agreement on Trade-Related Aspects of Intellectual Property Rights (TRIPS), there has been little emphasis given to assessing the implications of this amendment. The purpose of this review is to summarize the health and economic impact of patent protection, with a focus on the experience of Thailand.

**Methods:**

A review of national and international empirical evidence on the health and economic implications of patents from 1980 to 2009 was undertaken.

**Results:**

The findings illustrate the role of patent protection in four areas: price, present access, future access, and international trade and investment. Forty-three empirical studies were found, three of which were from Thai databases. Patenting does increase price, although the size of effect differs according to the methodology and country. Although weakening patent rights could increase *present* access, evidence suggests that strengthening patenting may benefit *future* access; although this is based on complex assumptions and estimations. Moreover, while patent protection appears to have a positive impact on trade flow, the implication for foreign direct investment (FDI) is equivocal.

**Conclusions:**

Empirical studies in Thailand, and other similar countries, are rare, compromising the robustness and generalizability of conclusions. However, evidence does suggest that patenting presents a significant inter-temporal challenge in balancing aspects of current versus future access to technologies. This underlines the urgent need to prioritize health research resources to assess the wider implications of patent protection.

## Introduction

Access to information that is generated through research and development (R&D) is a public good [1]. Because it is impossible to exclude people from using it, a price that reflects the actual cost of production cannot be charged. To address this, patents present a legal system that provides short-term exclusivity (or monopoly rights) over the production and sale of a specific product resulting from R&D. This allows the firm to sell at a price higher than would otherwise be the case, compensating the costs of R&D. However, there is some concern that the patent price is used to achieve ‘super-normal’ profits (profits in excess of those required to recoup R&D costs) at the detriment of wider access to patented products [2].

The implications of patenting spread further, as innovation, technology, and knowledge development are crucial drivers of economic development and technology transfer resulting from international trade and investment. The topic of patenting has found its way onto the global agenda due to the World Trade Organization (WTO)’s Agreement on Trade-Related Aspects of Intellectual Property Rights (TRIPS), which expanded the Western tradition of patenting to all members of the WTO. Under this Agreement, patent protection must be available for at least 20 years, must be without discrimination against the place of invention or origin of product, and must apply to both products and processes [3].

This has generated especially heated debate within the health community concerning the impact that patents may have on the price of and access to medicines, affecting both availability and affordability. However, arguments concerning patenting tend to take one of two sides: that patenting should be continually strengthened to encourage greater trade and investment, or that patenting should be weakened to ensure that medicines are as cheap as possible in the belief that this will ensure the greatest access for those in need. There is seldom, if ever, consideration of both sides when informing policy makers how to strike a balance between affordable medicines, both now and in the future, and trade and investment. For instance, whilst continually strengthening patenting will likely lead to higher prices, thus further reducing access, weakening patenting may stifle long-term access since pharmaceutical companies might be reluctant to introduce new medicines into the market, and foreign investors may look to invest in other countries where there is better protection of their products. In order to determine the appropriate balance in policy (such as the use of TRIPS-flexibilities), it is important to establish: (i) the impact that patent protection has on price; (ii) the impact that price has on current and future access; and (iii) the impact that patents have on innovation in national and international settings. This paper addresses these issues through a review of the literature concerning these areas, with a specific focus on Thailand.

### Patenting and Thailand

Thailand is a lower-middle-income economy with a 2007 per capita Gross National Income of US$3,400 and a total population of 63.3 million [4]. Health services are provided by both public and private insurance schemes, with public insurance schemes covering 97% of the population. In 2007, 72% of national health expenditure was financed by public expenditure [5]. The prices of medicines in Thailand are set mainly by market competition, with no policy related to price regulation [6]. In 1979, Thailand's Patent Act (B.E.2522) established the first legal protection for inventions in the country, although only process patents for pharmaceuticals were originally covered. This Act was revised in 1992 to introduce product patent protection for pharmaceutical products (13 years ahead of TRIPS compulsory compliance) [7].

The justification to amend the Thai patent law was to interest multinational companies to invest in Thailand. The other expected benefit of restricted patent protection in medicines is that this could increase domestic capability and strengthen the local pharmaceutical industry by the transfer of new technologies into the country [8], as the aim of a patent is to encourage technology transfer. Although technology diffusion can take place through a variety of channels that involve the transmission of ideas and new technologies—such as the imports of high-technology products, adoption of foreign technology and acquisition of human capital through various means—Foreign Direct Investment (FDI) was claimed as the most important channel for technology transfer [9,10].

This revision coincided with the rise of HIV/AIDS as a major health problem together with concern over the rising costs of anti-retrovirals (ARV). For instance, 200 mg (100 capsules) of original efavirenz sold in 2006 at 3,192 baht per bottle, while a generic equivalent was available at 1,292 baht [11]. A similar concern was expressed over other new medicines, which were not covered by the National Health Insurance system due to their high price [12]. The Sub-committee on selecting essential medicines under the National Health Insurance schemes therefore proposed compulsory licensing for seven patented medicines during 2006–2008 [13].

There were reactions from pharmaceutical companies. For instance, Abbott Laboratories withdrew its registration application for 10 new medicines in protest of the government use license on its product. These reactions were not confined to the pharmaceutical industry. In 2007 the Office of the United States Trade Representative elevated Thailand’s ranking from the Watch List to the Priority Watch List, indicating concerns over deficiencies in Intellectual Property Rights (IPR) protection and enforcement [14], and announcing that privileges under the Generalized System of Preferences would be removed for three Thai products: gold accessories jewellery, polyethylene terephthalate, and flat screen television sets [15].

**Figure 1:**
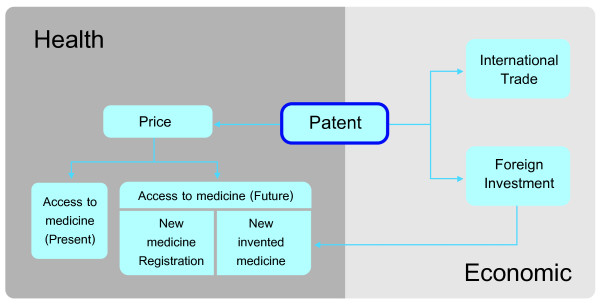
Conceptual framework of the implications of patenting on health and economic.

From the experience of pharmaceutical patent protection and associated policies during 1992–2008, it is apparent that patent protection has both health and economic consequences. A conceptual framework illustrating the broad implications of patent protection is illustrated in Figure 1. Patent protection affects the price of pharmaceuticals whereby price is a component in determining affordability and therefore access to existing medicines and industry investment in introducing or developing new medicines. A higher price would hinder access, but stimulate the development of new medicines through a higher R&D budget enabling patients to benefit from access to new medicines in the future. Patent protection is also accompanied by foreign investment in domestic facilities for the production of pharmaceuticals. Finally, as indicated above, there are wider trade relationships that may be affected by patent decisions, and which are not related to medicines at all.

## Methods

To ensure a manageable review, inclusion criteria covered the study scope and the type of study.

### Study scope

Studies which assessed the implications resulting from patent protection for pharmaceutical products and/or processes, especially:

· the benefit and cost of patent protection for the pharmaceutical industry

· the interplay between patents and the affordability and availability of medicines

· the relationship between patents and new drug development

· the implications of patent protection on the wider economy, i.e. trade or foreign direct investment.

### Types of studies

· Thai or English publications

· Research affecting any country

· Research articles, working papers or reports

· Research based on quantitative data (direct observation or experiment)

The databases searched covered both health and economic literature: Pubmed, Embase, Global health, International Bibiography of Social Sciences (IBSS), ABI/INFORM, and Econlit. The selected Thai databases were the Health System Research Institute database, Journal of Health Science, Thai thesis database, The Thailand Research Fund, Thai Journal Citation Index Center, and the Research Library of the National Research Council of Thailand. Given the specific case study setting of Thailand, the review of Thai literature also included unpublished (grey) literature such as research reports, Master’s dissertations, or Ph.D. theses. The dates of the published studies (1980–2009) were set so as to ensure the inclusion of all work conducted when the requirement of global standard patent protection was needed as, since the 1980s, intellectual property has became an important business tool, and new internationally-agreed trade rules for intellectual property rights were seen as a way to cope with the international economic tension [16].

The keywords employed in search queries covered five areas of interest: (i) patent policy, (ii) health, (iii) price, (iv) access, and (v) economy. In each area, the related keywords are identified in Table 1.

**Table 1:** Keywords related to a research area

**Areas**	**Keywords**
Patent policy	Intellectual property rights, Patent protection, Patent TRIPS, TRIPS Plus, TRIP flexibility, Free trade agreement Trade agreement
Health	Public Health, Health, Drug, Pharmaceutical, Medicine
Price	Price, Budget, Profit, Revenue
Access	Access, Afford/Affordability, Available/Availability
Innovation	Innovation, Research and development/R&D, Incentive New molecular entity, Invention
Economic growth	Investment, Trade/International Trade, Foreign direct investment/FDI, Economic growth

## Results

Initial searches resulted in 4,012 abstracts, of which 61 were from Thai databases. Of these, 43 passed the inclusion criteria, including only three from the Thai databases. Papers were categorised into: (i) role of patent on price, (ii) role of patented price on present access, (iii) role of patented price on future access, and (iv) role of patent on international trade and investment. Tables 2, 3, 4, 5, and 6 provide a brief summary of these empirical studies.

**Table 2:** Empirical studies of the implications of patenting on price

**Authors/ Ref. no.**	**Period**	**Setting (Country/medicines)**	**Objectives**	**Model**	**Method**
Watal (2000)/[17]	1985-1992	India, 22 patentable medicines in mailbox (varied in wide therapeutic areas)	The effect of product patents, price control and compulsory licensing on medicine prices and welfare.	Demand function estimation	Comparing effects from different demand functions—the constant elasticity demand and the linear demand function—and estimating price as a composite of the demand function.
Fink (2000)/[18]	1992	India, two therapeutic groups: quinolones and synthetic hypotensives	The impact of product patents on medicine price and pharmaceutical company’s profit	Demand function estimation	Modeling a demand function as a two-stage decision-making process (chemical entity and brands under that chemical entity). Then estimating price and profit under each substitution elasticity among chemical entities and among brands.
Boersma et.al. (2005)/[19]	1996 to 2001	The Netherlands, three medicines whose patents expired between 1996 and 2001	To observe price and share prior to and after patent expiration	Observational study	Trend analysis of volumes and price (measured as defined daily doses (DDD) prior to and after patent expiries were calculated
Suh et al. (2000)/[20]	1984-1987	USA, 35 chemical entities whose patents expired between 1984 and 1987	The effect of generic medicine entry on price after patent expiration	Regression analysis	Collecting descriptive statistics of price after patent expiration and analysing the influential factors affecting price, which are number of multiple-source medicines, market growth, market size, profitability, severity of illness, duration of treatment, and number of years after patent expiration.
Magazzini et al. (2004)/[21]	July 1987-December 1998 (Quarterly data)	USA, UK, Germany, and France, all medicines whose patents expired within the study period	Price and determinant of price after patent expiry	Regression analysis	Collecting descriptive statistics of prices before and after patent expiration. Using regression of the price with control of market share of the patented products, market size, % of sales to the hospital segment, the average market growth, the number of brand names, ratio of the average price of original products, etc.
Grabowski and Vernon (1992)/[22]	1983-1986	USA, 18 expired patent medicines	The pricing and competitive behaviour after patent expiration	Regression analysis	Using descriptive statistics of price index of the overall market, original medicine, and generic medicine. Using regression of the determinant of generic entry.
Griliches and Cockburn (1994)/[23]	1987-1990	USA, two anti-infective drugs: cephalexin and cephradine	The pricing and competitive behaviour after patent expiration	Observational study	Calculating the aggregate price indexes for a simple two-goods world where consumers buy either the brand or the generic version of a drug.
Borrell (2007)/[26]	1995-2000	14 antiretroviral therapy medicines in 34 low and middle income countries.	The impact of patents on medicine prices across developing countries	Regression analysis	Developing a price function as a composite function of the number of medicines in patent and non-patent regimes, number of generics after patent expiration, number of doses per day, efficacy, adverse reactions, and number of years in the US market.
Supakankunti et. al. (1999)/[27]	1987-1998	Thailand, six therapeutic categories were chosen to represent the patented market	The effect of new patent law on price	Observational study	There were no patented medicines so these medicines were selected by other criteria. Descriptive statistics were used to report the price movement or trend of the real price and nominal price of branded and generic medicines.
Limpananont et. al. (2004)/[28]	2001-2004	Thailand, antiretroviral therapy medicines	Price differences of patented and generic medicines	Observational study	Comparing and calculating the price ratio of patented and generic DDD prices
Jones et al. (2001)/[29]	1981-1994	Canada, 82 medicines from the British Columbia Pharmacare Programme.	The impact of the Canadian Patent Act in 1987	Regression analysis	Using descriptive statistics of prices before and after 1987 and log regression of generic market share, one factor, to predict market price.
Challu (1995)/’ [30]	1987-	Italy, 38 medicines	The impact of the 1978 patent law change	Observational study	Comparing new drug prices in Italy before and after the 1978 patent law. Using US prices as a reference.

**Table 3:** Empirical studies of the implications of patenting on present access

**Authors/Ref. no.**	**Period**	**Setting (Country/medicines)**	**Objectives**	**Model**	**Method**
Akaleephan et al. (2009) [31]	2000-2003	Thailand, top 70 imported medicines.	The implications of the TRIPS-Plus proposal, and extension of patent life on price and access	Regression analysis and Modelling	It was assumed that the first medicine patent expired in 2003. Drug consumption and budget from using generic were estimated. This cost was then compared with increased cost from patent life extension.
Yamabhai et al. (2009) [32]	2006-2008	Thailand, 7 government use licensed medicines in ARVs, heart disease and anti-cancer	The implications of Thailand's government use licenses on health and trade and foreign investment	Regression analysis and Markov model	Estimating the increased no. of patients with access to government use license medicines from the current number of access and up to 5 years. The Markov model was used to simulate the heath impact. Trend analysis of export and foreign direct investment was employed.
Attaran (2004)/[33]	2003	65 low and middle income countries, 319 WHO essential medicines	How many medicines are under patent in low and middle income countries?	Survey	Surveying pharmaceutical companies and their patent agents to determine where and how patentable medicines in the essential list of the WHO are now patented in developing countries
Borrell (2003)/[34]	1995-1999	34 low and middle income countries, HIV/AIDS medicines	The impact of patent rights on medicine sales: reducing or increasing.	Modelling	Developing two simultaneous relationships: (1) the relationship between the likely entry decision across drug-country-year triplets and patents; and (2) the relationship between market coverage (i.e. mean coverage of patients with a specific ARV drug) and patents conditional on drug entry decisions and patent regime.

**Table 4:** Empirical studies of the implications of patenting on incentive to introduce medicine to market

**Authors//Ref.no.**	**Period**	**Setting (Country/medicines)**	**Objectives**	**Model**	**Method**
Mansfield (1986)/[37]	1981-1983	100 U.S. manufacturing firms from twelve industries including the pharmaceutical industry	The effect of patent protection on the rate of development and commercialization of inventions.	Survey	Surveying U.S. manufacturing firms in order to know the proportion of its inventions developed in 1981–83 that would not have been developed and or commercially introduced if it could not have obtained a patent.
Lanjouw (2005)/[39]	1982-2002	68 countries at all income levels and including all medicine launches over the period of study.	How patent rights and price regulation affect whether new medicines are marketed in a country, and how quickly	Probit model	Using probit models of the probability that a new medicine is launched in a given country within either two years or ten years of the medicine’s first appearance in the global market and a log-logistic hazard model of the time path of country launches

**Table 5:** Empirical studies of the implications of patenting on incentive to invent new medicine

**Authors/Ref.no.**	**Period**	**Setting (Country/medicines)**	**Objectives**	**Model**	**Method**
Grootendorst(2007)/ [42]	1988-2002	Canada, prescription medicine expenditure	The implications of patent policies (Bills C-22 and C-91) on medicine expenditure and on R&D activity	Modelling	1. Estimating the medicine expenditures as a function of year dummies and lagged public drug expenditures, while controlling for a vector of other covariates that could affect drug spending. 2.Estimating R&D expenditure whose patent policy changed as an influenced factor
Hughes et al. (2002)/[43]	2001	USA	The effect of patent termination on current and future patients	Modelling	From models developed by various scholars during 1987–2002, five step models were estimated:1) the effect of patent termination on total revenue, 2) the effect of total revenue on R&D budget, 3) the effect of R&D budget on new medicine development, 4) the effect of new medicine on life year and 5) life year in monetary term
Giaccotto C. et al. (2005)/[44]	1980-2001	USA	The effect of price control policy on number of new drugs	Modelling	Estimating the decreased R&D budget as a function of five main items (price, GDP, foreign sales, dummy variables representing the years for which the Kefauver-Harris amendment and the Waxman-Hatch Act). The value of forgone R&D was then used to calculate the number of forgone drugs by dividing with $802 million (cost of R&D per drug)
Colleen (2003)/[45]	1980-1990	USA, six compulsory licensing (CL) medicines	The rate of innovation activities of pharmaceutical companies after CL	Observational study	Observing the rate of patenting and other measures of inventive activity five years before and after CL

**Table 6:** Empirical studies of the implications of patenting on economic growth and/or foreign direct investment

**Authors/Ref. no.**	**Period**	**Setting**	**Objectives**	**Model**	**Method**
TDRI (2003)/[47]	2003	Thailand	The impact of Thai-US FTA on export and import	Computable General Equilibrium (CGE)	Estimating benefit from trade in goods and the benefit to the economy as a whole by matching the industries that have higher revealed comparative advantage (RCA) index
Ferrantino (1993)/[48]	1982	U.S. firm, U.S. affiliated in 45 countries	The effect of IPR on trade and investment flows	Gravity model	Using dummy (0/1) variables to reflect differences in national IPR protection schemes and control for economic risk (distance, phone, landlocked, colony and European countries), political risk (Paris Convention member, restriction to foreign firm, number of international memberships, duration of patent), labour costs, population and GDP, while dependent variables are total export, royalty fee, sales of affiliate
Markus and Penubarti (1995)/[49]	1984	28 manufacturing sectors across 77 countries	The effect of IPR protection on trade flows	Regression	Using an empirical model in which deviations of bilateral sectoral imports from anticipated levels are related to income, trade barriers, and patent laws
Braga and Fink (1999)/[53]	1989	89 countries from developed to least developed countries	The effects of increased protection on intellectual property	Gravity model	Using a gravity model of bilateral trade, foreign direct investment, and technology licensing and estimating the effects of increased protection on a cross-section of 89x88 countries. Using the index on national IPRs systems developed by Park and Ginarte (1996). Estimating the effects of explanatory variables (such as IPRs, GDP and population of both countries, geographical distance, a common border, language)
Pradhan (2007)/[51]	1970-2000	India	The effect of patent protection on pharmaceutical exports	Gravity model	Using a gravity model consisting of GDP of the importing country, distance, trading bloc dummy, price and exchange rate
Kondo (1995)/[52]	1976-1980	U.S. outward FDI in 33 countries	The effect of patent protection on FDI	Survey (for IPR index) and Multiple regression of FDI testing	Developing their own patent index including scope, patent life, and provision from weighted point survey firm. Then using control factors of GDP per capita, population, education, English language, GATT member and ICSID member.
Pfister and Deffain (2005)/[54]	1994-1995	The location choices of French firms in 17 developing countries	The role of the patent rights in the host country	A conditional logit model	The independent variable is the decision to invest in the countries. The independent variables are number of French competitors, number of subsidiaries, openness, GDP, GDP per capita, consumer price index, status of EU union, national R&D investment over GDP, education, democracy, corruption, patent protection index (Ginarte and Park index), and dummy variable of the exceeding patent protection index.
Fosfuri (2004)/[55]	four time periods:1981–1983, 1984–1987, 1988–1991, 1992–1996.	75 countries receiving investments in chemical plants during the period 1981–1996	The impact of IPRs protection compared with country risk on the determinants of international activity through wholly owned operations, joint-ventures and technology licensing,	OLS, Tobit and GLS random effect	Independent variables are income per capita, population, weighted distance of country, averaged years of schooling among the total population, (exports + imports)/GDP, global index of risk, composite index of risk (political, financial and economic), dummy variables for number of scientists and engineers per million of population, time fixed effect, and IPR index by Ginarte and Park
Nunnenkamp and Spatz (2004)/[56]	1995 and 2000	U.S. FDI and US FDI in industrial level in 166 countries	The relationship between IPR protection and overall FDI and by industry	Gravity model regression	Finding FDI determinants through a regression of FDI on GDP per capita, population, distance to U.S., the cost of living abroad, average years of schooling and IPRs index using Ginarte-Park for the year 1995 and World Economic Freedom (WEF) index for the year 2000. Testing the industry characteristics by adding industry dummies in the previous independent variable set.
Lee and Mansfield (1996)/[57]	1991	U.S. firms and investment in 14 developingcountries	The effect of IPR protection level on U.S. firm’s FDI and the role of IPRs protection in chemical industry	1. Survey for IPRs protection perception 2. OLS regression 3. Tobit model for chemical industry	Surveying perceived weakness of IPR protection from 94 US firms and developing two regression models to find the influence of IPR protection level for overall US FDI and level of technology transfer in the chemical industry. For OLS of overall US FDI, independent variables are weakness of IPR, size of market, with control for firm specific and country specific, IPR index, market size, dummy for Mexico, FDI in previous year, degree of industrialization, openness, and time dummy variables. For Tobit model from 14 US chemical industries, the independent variables are the percentage of firms that felt weakness of IPR protection, GDP, and dummy variables for firms, while the dependent variable is the percentage of firms that will invest in facilities for sales and distribution.
An et al. (2008)/[58]	1995 (for FDI or licensing) and 1994 (for exporting)	U.S. FDI in 52 manufacturing industries invested in 62 host countries	To examine the effect of strengthening IPR protection on the mode of technology transfer: exporting, FDI or licensing	A multinomial logit model of three mode of entry choices	The explanatory variables covering national characteristics, GDP, absorptive capacity, distance, cultural distance (English and index developed by authors), FDI fixed cost (economic freedom index), market capitalisation and investment cost index, and IPR index from Ginarte and Park 1990. The industry characteristics variables are industry R&D intensity and capital intensity (the ratio of total real capital stock to total industry sales).
Maskus (1998)/[59]	1989-1992	U.S. FDI in 46 countries	The effect of patent protection on U.S. patent applications filed in host country, total sales of foreign affiliates of U.S. parents, U.S. exports shipped to affiliates and total assets, foreign affiliates of U.S. parents	Seemingly Unrelated Regression	Estimating a simultaneous set of equations to capture these joint impacts, controlling for market size, tariff protection, the level of local R&D by affiliates, distance from the US, investment incentives (proportion of affiliates receiving tax concession numbers in host country and in any of the countries) and disincentives (proportion of affiliates that employ a minimum amount of local personnel no. in host country and in any of the countries.
Javorcik (2004)/[60]	1995	1,405 global firms investing in Eastern European countries	The impact of intellectual property protection on the volume of FDI	Survey and Probit model	A questionnaire of decision to invest in any country and mode of entry was developed. Using a Tobit regression of the decision and mode of entry on GDP per capita, population, corporate tax rate, legal effectiveness, corruption, privatization, openness, the overall progress in reform, effectiveness of the legal system, corruption level, privatization policies and openness to trade. For testing the mode of entry, the author included firm specific variables such as firm sales, R&D outlays as a percentage of net sales, selling, general & administrative expenses as a percentage of net sales, the number of four-digit SIC codes describing a firm’s activities and a dummy variable of each investor’s regional experience in the region before 1989.
Du et al. (2008)/[61]	1993-2001	6,288 US firms investing in various regions of China	The impacts of four economic institutions variables, including property rights protection, the degree of government intervention in business operations, the degree of government corruption and contract enforcement, on the location choice of foreign direct investment	Discrete choice model	A survey was conducted of private enterprises in China to create three indexes which are the degree of government intervention in business operations, the degree of government corruption, and contract enforcement. The other concerned variables are the agglomeration, dummy for presence of US Embassy or Consulates and dummy for government promotion policies, wages, infrastructures (length of highway per square kilometre in a region), and education (percent of higher education students in the region). IPR index is the logarithm of the patent per capita approved number.
Kawai (2009)/[62]	1998-2006	1,839 Japanese manufacturing firms investing in China	The determinants of Japanese manufacturing firms’ location decisions in China	A conditional logit model	Empirical models were developed and tested. The dependent variable is choice of investment (1 = Yes, 0 = No). The independent variables are natural logarithms of the number of Special Economic Zones, IPRs index, natural logarithm of the share of total investment in fixed assets by state-owned units in relation to total investment, GDP, labour costs, road infrastructure and natural logarithm number of Japanese manufacturers All explanatory variables are lagged by one year.
Seyoum (2006)/[63]	1990 and 1995	63 countries	The impact of patent protection FDI	The OLS regression	Using the set of independent variables which include patent index by Ginarte and Park (1997) and controlling other variables such as market size, GDP growth, exchange rates, population, corruption, unemployment, trade/GDP, scientists and engineers, GDP growth
Lesser (2002)/[64]	1998	FDI in 44 developing countries	The effects of stronger IPR protection in the areas of imports and Foreign Direct Investment (FDI)	Multiple regression	The variables includes income per capita, past FDI, exchange rates, tariffs, the proportion of previous year FDI to GNP of pervious year, and the degree of industrialization. A new index was developed that uses membership in international treaties to measure the scope and efficiency of IPR.
Park and Ginarte (1997)/[65]	1960–1990	60 countries from developed to least developed countries	The impact of IPR protection on economic growth (GDP growth)	Regression	Creating an IPR index and estimating a system of equations to identify the effect of IPR protection and other national characteristics on economic growth such as R&D activity, investment, and education
Athukorala and Kohpaiboon (2006)/[66]	1990-2001 (three-year intervals)	168 US-based MNEs that have invested internationally (42 countries)	The determinants of the international location of R&D activity by foreign affiliates of US-based MNEs	Regression analysis	Included control variables are real GDP, distance, percentage of domestic sales in total affiliate sale turnover, technology intensity index, R&D personnel per million population, wages of technical personnel, tax incentives for firm-level R&D activities, intellectual property rights index (from World Economic Forum, Global Competitiveness Report), capital stock of US firms, an index of R&D potential of output mix, dummy variables for developing countries other than NICs, newly industrialized countries in East Asia, financial crisis dummy, and vector of time dummy variables
Blyde and Acea (2003)/[67]	1985, 1990 and 1995	The sources of FDI are 19 OECD countries and 40 countries as the recipients of FDI, 8 of which are from Latin America.	The inflows of foreign direct investment of Latin America and developing countries after TRIPS	The gravity model	The independent variables are GDP per capita, population, dummy of common language, past colonial links and region, distance between countries, Ginarte-Park IPR index
Supakankunti et al. (2001)/[70]	1988-1998	Thailand	The impact of patent law change in 1992 on FDI in Thailand	Observation	Providing the trend of FDI for industry in general and specifically for the chemical industry in Thailand

### Role of patent on price

Twelve studies, including two Thai studies, looked at the effect of patents on price. Most of these studies focused on the patent expiration effect in the USA. Patent protection appears to increase price by around 26%-277% depending on which of the three estimation approaches is used.

The first approach uses elasticity of demand to calculate price. This has been used to estimate the likely effects of patents on the price of medicines not currently under patent protection, and to extrapolate to a situation of those medicines being under TRIPS obligations. Using this methodology, Watal (2000) showed that all patentable medicine prices in India would increase by a mean of 26% with linear demand and 242% with constant price elasticity of demand [17]. Similarly, by accounting for different products through trademarks and advertising, Fink (2004) used this approach to estimate that prices would increase by 30–277% if these medicines came under patent protection [18].

This approach would be useful if the price elasticity of demand is known and correct. For the pharmaceutical market, the consumption decision commonly involves participation by a physician and a third party payer (government or hospital committee). The consumer may or may not play some part in the price payment, depending on a country’s specific regulatory and reimbursement regimes. The pharmaceutical market’s demand function is thus often distorted, and a model based on price elasticity of demand might not present a real world situation of the complexity of the pharmaceutical market.

Second, the observation of price before and after patent expiration is used to infer the price effect of patent protection. Boersma et.al. (2005) illustrated that—when there is no patent protection—prices generally fall by 50–70% [19]. Suh et al. (2000) showed a decline to approximately 30% of the original price three years after patent expiration [20]. Also, Magazzini et al. (2004) showed that the price index decreased three years after patent expiration by approximately 20% in Germany and France while the UK price index was stable [21]. Conversely, two US studies by Grabowski and Vernon (1992) and Griliches and Cockburn (1994) showed that—following generic entry—an original product can have an increase in price of 7% and 11% after one year and two years respectively [22]. Another study showed a 60% price increase three years after expiration, while the generic price decreased by 30% [23]. These increases may reflect increased advertising intensity once the market protection of patenting has expired.

However, the effect in each country will differ since each nation has a different health system in terms of medical tradition, policy for financing and supporting generic entry, and brand royalty of physicians, pharmacists and customers. The marketing strategy also differs among pharmaceutical companies, who often spend more heavily on the intensity of advertising once the patent has expired, which could explain at least some of the post-patent price increase. It appears that medicine prices, in general, depend on several supply and demand factors. For example, therapeutic advantage and number of substitutes are both significant price determinants; as the number of substitutes increased in one study from one to two, there was an average 38% decline in the ratio of the new drug price to the average existing market price [24]. Kanavos and Vandoros (2011) also found that product age has a significant and negative effect on prices [25].

Third, studies perform regression analysis of factors influencing medicine prices, of which patent is one such factor. Borrell (2007) estimated patent impact on price in 14 ARV molecules in 34 low- and middle-income countries which have different patent regimes, where patent was eligible or ineligible between 1995 and mid-2000. This showed that combination therapy containing at least one patented medicine is on average priced 70% higher than combination therapy containing only generic alternatives. Combination therapy containing at least one original medicine is priced 16% higher than local copies even when it is introduced in to no-patent regimes [26].

In Thailand, the introduction of product patent protection in1992 seems to have had no effect on the price of patentable medicines that were in the market before 1992 [27]. Rather, the effect appears to concern only those patented medicines which were introduced subsequently. For example, one study compared the price of patented and generic HIV/AIDS medicines from 2001–4 and found that the patented price was approximately 1.5-3 times higher than the generic price in 2001 [28].

The experiences in Canada and Italy reflect the situation in Thailand. A study of the impact of the 1987 Canadian Patent Act, which extended the period of protection from seven to ten years while also allowing the generic industry to implement compulsory licensing, found that after 1987, medicine prices increased relative to pre-1987 prices [29]. Similarly, after a patent law in Italy came into effect in 1978, new medicine prices were 163% higher than new drug prices before 1978 [30].

### Role of patented price on present access

Empirical evidence directly linking patented price and access is rare. Most studies describe how patenting increases prices (as above) and then *assume* that price affects access, but there is a lack of direct association between the extent of price increase and the extent of changes in access, controlling for other influence factors. As shown in Table 3, there were four studies found concerning this issue, two of which are in the Thai setting.

Akaleephan et al. (2009) examined the effect of patent life extension from a TRIPS-Plus proposal on access to medicines. They illustrated the drawbacks of extending the period of protection by showing that the availability of generics would help to save 105% of actual government expenditure, and accessibility would increase by 54% [31]. In addition, after compulsory license introduction in Thailand, the price of generic medicines was about 3–38% of their original price. As a result, there were approximately 8,000 extra patients utilizing EFV, and it is estimated that the increased number of patients with access to EFV will be 17,959 in five years [32].

Conversely, Attaran (2004) suggested that the main obstacles are associated with the country’s socio-economic status, such as the lack of manufacturing capacity or poor health care systems [33]. His survey results show that only 17 from a total of 319 medicines on the WHO Essential Medicines List are protected by patents. In addition, Borrell and Watal (2003) showed that switching all medicines under a patent regime to a no patent regime globally would have increased the percentage of AIDS patients with access to new medicines from 0.88% to 1.18% [34]. However, with reference to individual countries, the findings suggested different magnitudes of impact. For example, in Thailand where most of the relevant medicines were under patent, it was estimated that around 10,000 additional prescriptions would be prescribed if all patents were waived, generating an increase in access of some 50%.

### Role of patented price on future access

#### Incentive to introduce medicine to market

Pharmaceutical companies may refuse to market new medicines in response to weak national patent policy [35,36]. Mansfield (1986) estimated that 65% of products would not have been introduced if patent protection could not have been obtained [37]. While patents make local markets more attractive, multinationals may delay or avoid launching medicines in lower-priced countries because they are concerned about the implications for pricing in other markets [38]. For instance, Lanjouw (2005) determined the effects of patent policy and price control policy on market entry, and showed that extensive price control and process-only patent protection lowers the probability of having a new medicine in lower-income countries by 30% [39]. A brief summary of these two studies is shown in Table 4.

The model employed in these two studies was multi-country, from high-income to low-income countries. Although the results are more generalized, they sometimes mislead. Under some circumstances and model assumptions, patent protection has a positive effect for some countries, while under other circumstances it has a negative effect. Single country studies are particularly effective at maximizing their explanatory leverage by exploiting the availability of comparable units of analysis, whether over market or medicine characteristic variations within a country [40].

#### Incentive to invent new medicine

One implication of removing patent protection to gain increased current access is that this might result in patients foregoing the opportunity to receive a new medicine in the future, as it would not be discovered or developed [41]. There were four studies looking at this possibility, as shown in Table 5. Grootendorst (2007) illustrated that this clearly generates trade-offs between benefits now and in the future [42]. Indeed, heavily depending on assumptions, Hughes et al. (2002) estimated that for every dollar in consumer benefit realized from providing greater access to current medicines, future consumers would be harmed at a rate of three dollars in present value terms from reduced future innovation [43].

Giaccotto C. et al. (2005) investigated the role of price control on new medicine development, showing that price control policy in the USA during the 1980s resulted in forgone R&D investment of US$264-293 million, translating into 330–365 fewer new medicines, which is equal to one-third of all actual new medicines launched on the global market during that time period [44]. However, such studies lack a direct link between profitability and actual investment in R&D. They illustrate the effect of patents on profit and assume that this translates directly into R&D. Conversely, an observation of the innovation activities of pharmaceutical companies affected by compulsory licensing found that there was no uniform decline in the rate of medicine patenting and other measures of inventive activity by companies affected by compulsory licenses [45] Table 5.

### Role of patent protection on international trade and investment

With respect to the broader impact, in a Free Trade Agreement (FTA) between Thailand and the US, one of the 23 negotiation issues was TRIPS-Plus, which requires a higher level of intellectual property protection than existed in the TRIPS agreement [46]. Five studies focusing on the impact of patents on trade were found. Based on a Computable General Equilibrium model, it was estimated that the FTA would increase the export and import levels of Thailand by 3.4% and 4.7%, respectively [47]. This study also found, at the international level, that IPR protection had a positive influence on overall trade flows for both small and large developing economies [48-50]. These results are in line with other findings which show that patent protection had a positive impact on Indian pharmaceutical exports [51].

### Intellectual Property Protection and investment

Nineteen studies, including one Thai study, looked at the effect of patents on FDI. Most of these studies used regression to analyze the effect of IPRs on FDI. Additional variables are included in the regression to control the differences in country specific factors. Their main similarity is in comparing IPR risk along with economic risk and/or political risk. Proxy indicators were used to represent economic or political risk. Most of these focused on the role of the national patent protection policy to attract US investors.

Six empirical studies found that patent rights protection does not influence the location choices of foreign investors. Some regression analyses of FDI in the 1980s based on research by Ferrantino (1993), Markus and Penubarti (1995), Kondo (1995), and Braga and Fink (1999) found no significant link between IPR protection and FDI [48,52,53]. Using FDI data from the 1990s, the above results were again confirmed in the study of Pfister and Deffains (2005) who investigated the role of patent protection on the location choices of French firms investing in 17 developing countries [54]. In addition, the Fosfuri’s (2004)’s study, which focused specifically on the chemical industry and accounted for the differences of country characteristics, did not find that IPR protection played a significant role in fostering international activity [55].

However, some studies revealed that the volume of FDI in a country tends to be inversely related to the weakness of IPR protection. Five studies looking at the FDI determinants of US Multinational Enterprises (MNEs) were found. The results show that the strength of IPR protection has a significant positive impact on the U.S. FDI [56]. For example, a one percent rise in the perceived weakness of IPR protection would decrease the U.S. FDI in that country by 14%. In a sample of chemical firms, it was found that firms are likely to allocate their investment to sales and distributions and simple production activities rather than to manufacturing final products or to R&D facilities [57]. This is confirmed in another study [58], in which it was suggested that a one percent rise in the extent of patent protection would increase the U.S. investment in that country by 0.45% [59].

Javorick (2004) indicated that weak protection of intellectual property rights deters foreign investors in four technology-intensive sectors: (1) drugs, cosmetics and health care products; (2) chemicals; (3) machinery and equipment; and (4) electrical equipment. In addition, foreign investors, in all industries, tend to set up distribution facilities rather than engaging in local production in a country with weak IPR protection [60]. Four studies confirmed that MNEs prefer investing in the regions that have better intellectual property rights protection [61-63]. For example, a one point increase in IPR index would boost FDI by $1.5 billion [64].

Three studies using regression analysis yielded inconclusive results when analyzed in subgroups. Two studies showed the positive impact of strong national patent laws in developed countries, but showed a negative impact in developing countries [65,66] while another study revealed the converse results [67].

In terms of the pharmaceutical industry specifically, a strong patent system was found to have caused a considerable flow of investment into the American pharmaceutical industry [68]. However, some studies show a negative correlation between the levels of protection and foreign investment. This is supported by conclusions elsewhere that the exclusion of pharmaceuticals from patent protection was a significant factor leading Italy to become a base for the export-oriented production of generic medicines [69]. Supakankunti (2001) showed that in Thailand there has been little foreign investment in the pharmaceutical sector since the introduction of the strengthened patent law in 1992 [70]. It has been suggested that this is because foreign investors consider Thailand an unsuitable destination due to the insufficiency of well-trained human resources, technology, and equipment, as well as the inadequacy of the registration system for new medicines [8] Table 6.

In conclusion, there are a number of empirical investigations pointing to an uncertain relationship between patent and FDI distribution, which depends on the political and business risks of the country included, FDI sources, data from opinion surveys or secondary data, and the approach used to calculate the level of patent protection scale. The question of just how important patent protection is for FDI is still unsettled. Some evidence indicates that patents have had a positive impact on FDI overall and the pharmaceutical industry in particular, while other evidence suggests that weak patent protection of pharmaceuticals was the main factor in making the country a manufacturing base for these pharmaceutical companies. As both country-specific and regional factors influence the effect of patents on FDI, more regional and country–specific studies should be conducted in order to validate the findings of this study. As noted by Lesser (2002), the effect of IPR on FDI may only be possible on a country-by-country basis.

## Conclusions

The empirical literature provides answers to some important questions related to the impact of patents, although evidence remains largely inconclusive. With respect to patent impact on price, both Thai and international evidence confirm that patenting shifts prices up and has an effect on the price of the new registration of medicines. In terms of present access, international empirical evidence demonstrates that patent protection does not always impede access, whereas a Thai study suggested that implementing a limited patent life may actually increase access. As for future access, evidence suggests that strengthening patent policy in a given nation may speed up the time required for entry into the pharmaceutical market. Empirical models estimate that higher profits, from patents, would increase the number of new medicines to market through higher R&D budgets, enabling patients to benefit from access to new medicines in the future. Conversely, one observational study revealed that withdrawing exclusive rights by compulsory licensing might not have an effect on innovation in the future.

**Figure 2:**
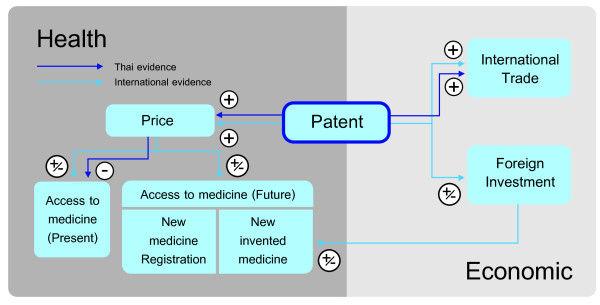
Availability of empirical evidence and direction of the relationship.

The evidence found from this review confirms that policy stimulating patent protection does have a positive impact on trade flows. In terms of FDI, evidence provides inconclusive results, both generally and specific to the pharmaceutical industry. Figure 2 summarizes existing evidence and the remaining gap, with an indication of the relationship found from this review.

The review revealed that little empirical research has been undertaken on the extent to which patent rights affect health and economic factors. With respect to health, the settings of the studies are very mixed across therapeutic areas and medicines. The literature generally shows that the size of impact varies wildly, depending on which methods are employed in the studies. Current evidence therefore makes it difficult for a country, such as Thailand, to come to a conclusion on advice to national policy makers who are to make decisions which trade off health or access impacts with wider economic issues. The high price of medicines may not be related to patent rights. Furthermore, price may not be related to access, either.

### Recommendations

The trade-offs between patent protection, current and future access to medicines, and related aspects of trade and investment, are still subject to debate, since empirical studies are relatively rare, especially in countries such as Thailand. This underlines the urgent need to prioritize health research resources to assess the implications of patent protection.

It is clear that evidence on the role of patent/price on access, especially with respect to non-communicable diseases, is scare, inconclusive, and problematic. This suggests a more holistic assessment is required which takes into account a country’s socio-economic status and health care system when estimating patent impact on access to medicine. The estimation of patent impact on technology transfer through FDI should be conducted on a country basis. In order to try and assess whether, on balance, a country is better off with patent policy related to health or not will require evaluating the implications for current and future access to medicines, and the wider national economy.

## Competing interest

We declare that we have no competing interest.

## Authors' contributions

IY designed the research methodologies and carried out the study. RD co-designed the methodologies, participated in discussions, and provided interpretations. The manuscript was written by IY and RS. All authors have contributed to, reviewed, and approved the final manuscript.
